# A method for dyadic cardiac rhythmicity analysis: Preliminary evidence on bilateral interactions in fetal–maternal cardiac dynamics

**DOI:** 10.1113/EP092532

**Published:** 2025-02-21

**Authors:** Diego Candia‐Rivera, Mario Chavez

**Affiliations:** ^1^ Sorbonne Université, Paris Brain Institute (ICM), Inria Paris, CNRS UMR7225, INSERM U1127, AP‐HP Hôpital de la Pitié‐Salpêtrière Paris France

**Keywords:** cardiac development, co‐regulatory mechanisms, heart rate variability, maternal–fetal interactions

## Abstract

Cardiac activity responds dynamically to metabolic demands and neural regulation. However, little is known about this process during pregnancy. Reports show occasional fetal–maternal heart rate couplings, but it has remained unclear whether these couplings extend to more complex oscillatory patterns of the heart rhythm. We developed a framework of time‐varying measures of heart rate and rhythm, to test the presence of co‐varying patterns in concurrent maternal and fetal measures (late pregnancy dataset, *n* = 10, and labour dataset, *n* = 12). These measures were derived from first and second‐order Poincaré plots, with the aim to describe changes in short‐ and long‐term rhythmicity, but also the dynamic shifts in acceleration and deceleration of heart rate. We found episodes of maternal–fetal co‐varying patterns of cardiac rhythm in all the measures explored, in both datasets (at least 90% of the dataset presented a significant maternal–fetal correlation in each measure, with *P *< 0.001), with dynamic delays suggesting bilateral interactions at different time scales. We also found that these couplings intensify during labour (test between late pregnancy vs. labour datasets, *P *< 0.0015 in all second‐order Poincaré plot‐derived measures). While most literature suggests that the fetal heart responds to maternal breathing patterns or contractions, we propose the possibility that the fetal heart may also have a signalling function in the context of co‐regulatory mechanisms and maternal inter‐organ interactions. Understanding these complex visceral oscillations in utero may enhance the assessment of a healthy fetal development.

## Abstract (highlights)

- **What is the central question of this study?**
  Do fetal and maternal cardiac rhythms exhibit co‐varying patterns, and if so, do these patterns suggest bilateral interactions rather than passive fetal responses to maternal changes?
- **What is the main finding and its importance?**
  The maternal and fetal hearts engage in bilateral interactions, with delays in rhythm fluctuations. These cardiac interactions intensify during labour. The study suggests that fetal physiology plays an active role in inter‐organ communication, emphasizing its significance in healthy fetal development.

## 1 INTRODUCTION

The autonomic nervous system plays a critical role in regulating physiological processes, which are typically observed as changes in electrocardiogram waveforms, heart rate and rhythm, also termed heart rate variability (Holzman & Bridgett, 2017). While the importance of cardiac biomarkers in capturing regulatory mechanisms is known (Levy & Martin, 1981), the specifics of maternal–fetal cardiac biomarkers remain unclear.

Parallel changes in cardiac dynamics can be measured from concurrent maternal–fetal electrocardiogram recordings. Previous studies have reported occasional couplings of maternal and fetal heart rates (Nichting et al., 2023), and the influence of maternal respiration (DiPietro et al., 2023; Van Leeuwen et al., 2009) and uterine contractions (Sletten et al., 2016) on fetal heart rate. However, the nature and significance of these interactions are not well understood. Beyond simple heart rate couplings, the way different fetal and maternal cardiac rhythms converge and interact has not been thoroughly studied.

Time‐resolved analyses of cardiac activity enable the assessment of couplings and, potentially, causal interactions. However, fetal and maternal ECGs are not directly comparable, as what is considered a fast heart rate for the mother is even faster for the fetus (David et al., 2007; Van Laar et al., 2008). Therefore, individual specific descriptors are necessary to properly study parallel fetal–maternal cardiac activity. To investigate maternal–fetal cardiac dynamics, we used time‐varying measures of heart rate and rhythm that account for individual‐specific changes in cardiac rhythmicity based on the Poincaré plot (Candia‐Rivera et al., 2025), and propose new measures to quantify dynamic heart rate acceleration and decelerations, based on the second‐order Poincaré plot. We sought to determine whether the maternal and fetal counterparts correlate and if there are delays suggesting causal interactions. Six descriptors of measures of heart rate and rhythm were examined on 20 min‐long resting state recordings during the 32–42nd gestation week (pregnancy dataset) and on 5 min‐long recordings during labour (labour dataset) (Matonia et al., 2020).

**FIGURE 1:**
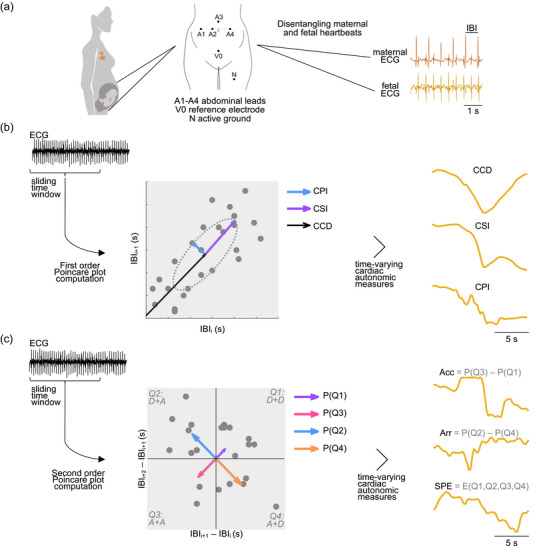
Methodological pipeline. (a) Maternal and fetal ECG were recorded simultaneously. Heartbeat detection at the R‐peak of the cardiac cycle allowed the computation of IBI series. (b) The variability of IBI over time illustrated using a first‐order Poincaré plot, which shows changes between successive IBIs. From the Poincaré plot, the baseline CCD, CSI, and CPI were extracted. (c) The variability of IBI over time illustrated using a second‐order Poincaré plot, which shows changes between three consecutive IBIs. From the second‐order Poincaré plot, cardiac acceleration–deceleration balance (Acc), arrhythmic behaviour (Arr) and SPE were obtained. CCD, cardiac cycle duration; CPI, cardiac parasympathetic index; CSI, cardiac sympathetic index; ECG, electrocardiograms; IBI, interbeat interval; SPE, second‐order Poincaré plot entropy.

The Poincaré plot (Figure 1) is a method used to describe the variability of successive changes in the duration of interbeat intervals (IBI) (Woo et al., 1992). Time‐resolved measures of heart rate and rhythm were derived from the Poincaré plot, which have been reported to capture the complex manifestations of sympathetic and parasympathetic activations. Using these descriptors, we explored the complex interactions in maternal and fetal cardiac activities.

Our research introduces methodologies for characterizing heart rate and rhythm and provides new insights into maternal–fetal cardiac autonomic interactions. These methodologies may have significant implications in future research on prenatal monitoring, with respect to the role of visceral oscillatory activity in utero, encompassing potential influences in perceptual processing, sensorimotor transformations and early cognitive development (Corcoran et al., 2025, 2023).

## 2 METHODS

### 2.1 Ethical approval

All human recordings were collected and monitored by qualified and trained medical staff, as part of research projects at the Department of Obstetrics and Gynecology of the Medical University of Silesia in Katowice, Poland. The research was approved by the competent University Bioethics Committee (IRB approval number NN‐013‐345/02). Participants provided a written consent to participate in the study, as required by the *Declaration of Helsinki*.

### 2.2 Study cohorts

This is a retrospective analysis on open access datasets of concurrent maternal–fetal electrocardiograms (Matonia et al., 2020). The pregnancy dataset (*n* = 10) was recorded between the 32nd and 42nd week of pregnancy, where a single recording of 20 min was obtained per participant. The labour dataset (*n* = 12) was obtained in the active labour stage (approximately 24 h before delivery), occurring between the 38th and 42nd week of gestation, where a single recording of 5 min was obtained per participant (Matonia et al., 2020). Annotations regarding the timing of uterine contractions were not available. During the pregnancy recordings, participants took a semi‐sitting position leaning slightly to the left, could talk freely and reaccommodate their position if required for comfort.

The KOMPOREL System (ITAM, Krakow, Poland) was used for monitoring the bioelectrical activity of fetal hearts. The system consists of a signal recorder module and a portable computer. The recorder module enables simultaneous acquisition of four signals from the maternal abdominal wall as well as one fetal ECG signal directly from the fetal head (for labour dataset only). The recorded analog signals are digitized with a 16‐bit resolution at 500 Hz sampling frequency for abdominal signals and 1 kHz for a direct fetal ECG.

The electrode configuration includes four measuring electrodes (A1–A4) evenly placed around the patient's navel. These electrodes record signals relative to a common reference electrode (V0) located above the pubic symphysis (Figure 1a). Additionally, a common‐mode reference electrode with an active‐ground signal is placed on the participants' left leg. This configuration was determined under a compromise between ease of application and the development of an effective maternal ECG suppression method to ensure high‐quality fetal ECG signals. Standard Ag/AgCl electrodes (Red Dot 2271; 3M, Maplewood, Minnesota, USA) were used during the monitoring, and the top layer of the skin was gently cleaned with conductive paste (Red Dot Trace Prep 2236; 3M, Maplewood, Minnesota, USA). The direct fetal ECG signal was recorded using a sterile spiral electrode (AB 15133C; CETRO, Bauru, Brazil) placed on the fetal head. All monitoring sessions were conducted by qualified medical staff and an experienced biomedical engineer to ensure proper signal acquisition (Matonia et al., 2020).

### 2.3 Data preprocessing

All signals were filtered to suppress power and slow‐changing interferences. A simple filter with multiple notches, located every 50 Hz, was used. The first cutoff frequency is equal to approximately 5 Hz, which assures effective suppression of low frequency noise. The top‐band between 45 Hz and 55 Hz results in successful elimination of the powerline interference.

A detailed description of the data preprocessing can be found in the original dataset paper (Matonia et al., 2020). In brief, abdominal signals were preprocessed to suppress the dominant maternal ECG. For this, the maternal ECG pattern was created by averaging continuous ECG fragments to create a QRS complex template (Matonia, Jezewski, Horoba, et al., 2006, Matonia, Jezewski, Kupka, et al., 2006). Maternal ECG suppression was performed in all the abdominal leads and the highest‐quality fetal ECG signal was selected, based on the signal quality index obtained from the autocorrelation function assessing the signal's quasi‐periodicity (Matonia, Jezewski, Horoba, et al., 2006, Matonia, Jezewski, Kupka, et al., 2006). Direct fetal ECG were cleaned using a normalized matched filtering to reduce sensitivity to interference. This was complemented by decision rules that minimize a cost function to predict the duration of consecutive fetal heart cycles. (Kotas et al., 2010).

Filtered signals were analysed with MATLAB 2022b (MathWorks, Natick, MA, USA). Heartbeats were automatically detected using an algorithm based on the Pan–Tompkins method (Pan & Tompkins, 1985). Ectopic heartbeats were automatically identified by detecting peaks in the IBI derivative, and manually corrected. The detection was visually compared to those registered manually by clinical experts. Finally, an automated correction of IBIs was performed, based on the Lipponen method (Lipponen & Tarvainen, 2019).

### 2.4 Data analysis

IBI series were constructed based on the R‐to‐R‐peak durations. A Poincaré plot was used to depict the fluctuations in the duration of consecutive IBI (Brennan et al., 2001). This method typically depicts these changes in an ellipsoid‐shaped distribution (Figure 1b), which can be analysed geometrically. The descriptors are as follows:

1. Cardiac cycle duration (CCD): This quantifies the distance from the origin to the center of the ellipse, indicating the baseline CCD, on top of the changes in heart rate variability (Sacha et al., 2013). A decrease of the CCD indicates sympathetic activations, and an increase indicates parasympathetic activations (Task Force of the European Society of Cardiology the North American Society of Pacing, 1996).
2. Cardiac Parasympathetic Index (CPI): This represents the minor axis of the ellipsoid, with its variability reflecting the fast fluctuations in heart rate variability associated with parasympathetic tone (Candia‐Rivera et al., 2025).
3. Cardiac sympathetic index (CSI): This represents the major axis of the ellipsoid, with its variability reflecting the slow fluctuations in heart rate variability associated with sympathetic tone (Candia‐Rivera et al., 2025).

The time‐varying fluctuations of the distance to the origin and the ellipse ratios were computed with a sliding‐time window T:

(1) $$\mathrm{CCD}\left(t\right)=\sqrt{\mathrm{mean}{\left(\mathrm{IB}I_{i,\text{…},n-1}\right)}^{2}+\;\mathrm{mean}{\left(\mathrm{IB}I_{i+1,\text{…},n}\right)}^{2}\;}$$

(2) $$\mathrm{CPI}\left(t\right)=\sqrt{\lambda_{\Omega_{t}}\left(1\right)}$$

(3) $$\mathrm{CSI}\left(t\right)=\sqrt{\lambda_{\Omega_{t}}\left(2\right)}$$

where $\lambda_{\Omega_{t}}$ is the matrix with the eigenvalues of the covariance matrix of $\mathrm{IB}I_{i,\text{…},n-1}$ and $\mathrm{IB}I_{i+1,\text{…},n}$, with $\Omega_{t}:\;t--T\;\leq \;t_{i}\;\leq \;t$, and n is the length of IBI in the time window $\Omega_{t}.$ *T* was defined at 15 s, as per previous simulations (Candia‐Rivera et al., 2021, 2025). The computation step was defined by each heartbeat timing and the final time series were evenly interpolated at 4 Hz. The eigenvalues were computed with a robust approach that computes the covariance matrix using a shrinkage covariance estimator based on the Ledoit–Wolf lemma for analytic calculation of the optimal shrinkage intensity (Schäfer & Strimmer, 2005).

The other descriptors are based on the second‐order Poincaré plot (Babloyantz & Maurer, 1996), which depicts IBI variability in groups of three consecutive IBIs (Figure 1c). The first quadrant of this plot shows the cases of two consecutive heart rate decelerations, while the third quadrant shows two consecutive accelerations. The second and fourth quadrants indicate cases where a deceleration is followed by an acceleration, and vice versa, respectively. From this, we conceived three additional descriptors:

1. Heart rate acceleration–deceleration balance (Acc): This is quantified by computing the difference between the dispersion of the first quadrant and third quadrant. This measure estimates the predominance of cases with two consecutive heart rate accelerations compared to cases with two consecutive decelerations within a defined time window. Since heart rate accelerations can be attributed to either activations in the sympathetic nervous system or deactivations of the parasympathetic counterpart (Stavrakis et al., 2020), we propose Acc as an alternative measure of the predominance of sympathetic activity over the parasympathetic one. Existing measures, such as the low frequency (LF)/high frequency (HF) ratio, proposed to quantify this predominance have faced significant criticism due to their lack of demonstrated specificity (Billman, 2013; von Rosenberg et al., 2017). Therefore, Acc still requires validation as a reliable measure of sympathetic tone predominance over parasympathetic tone and has yet to be demonstrated as an appropriate alternative to the LF/HF ratio.
2. Heart rate arrhythmic behaviour (Arr): This is quantified by taking the difference in dispersion between the second and fourth quadrants. This measure estimates the presence of consecutive accelerations and decelerations within a defined time window. While it is sometimes referred to as heart rate turbulence in the context of premature ventricular contractions (Bauer et al., 2008; Cygankiewicz, 2013), it can also be observed without these contractions. The physiological origin of this arrhythmic behaviour can be attributed to parallel sympathetic and parasympathetic activations, resulting in low magnitude arrhythmia (Stavrakis et al., 2020), or either sympathetic or parasympathetic activations depending on the time scale (Pan et al., 2016), often referred to as parasympathetic shifts for short‐term analyses (Campana et al., 2010; Pan et al., 2016).
3. Second‐order Poincaré plot entropy (SPE): This is defined as four states, computed from the proportion of points in each of the quadrants. This measure estimates the degree of changes in heart states, from consecutive accelerations to consecutive decelerations, or the appearance of short periods of heart rate turbulence, within a defined time window. In this context, these changes could be attributed to both sympathetic and parasympathetic activations (Pan et al., 2016; Stavrakis et al., 2020). Entropy would quantify the complexity on the transitions between heart rate acceleration, deceleration, and turbulence, potentially attributable to higher variability in sympathetic and parasympathetic activations or deactivations, although unspecific. For example, in brain microstates studies – research focused on scalp patterns observed in electroencephalography – a higher entropy reflects greater complexity in state transitions (von Wegner et al., 2024).

The time‐varying fluctuations of the second‐order Poincaré plot were computed with a sliding‐time window, as shown in Equations (4), (5) and 6. These measures are based on the number of data points present in each quadrant:

(4) $$\mathrm{Acc}\left(t\right)=P_{\Omega_{t}}\left(Q_{3}\right)-P_{\Omega_{t}}\left(Q_{1}\right)$$

(5) $$\mathrm{Arr}\left(t\right)=P_{\Omega_{t}}\left(Q_{2}\right)-P_{\Omega_{t}}\left(Q_{4}\right)$$

(6) $$\mathrm{SPE}\left(t\right)=\sum_{i=1}^{4}\left(-\frac{L_{\Omega_{t}}\left(Q_{i}\right)}{N_{\Omega_{t}}}\log_{2}\frac{L_{\Omega_{t}}\left(Q_{i}\right)}{N_{\Omega_{t}}}\right)$$

where $P_{\Omega_{t}}\left(Q_{i}\right)$ is the dispersion of the quadrant, defined as 2 standard deviations of the Euclidean distance to the origin of all the points part of the quadrant $Q_{i}$. $L_{\Omega_{t}}\left(Q_{i}\right)$ indicates the number of points in the quadrant $Q_{i}$, and $N_{\Omega_{t}}$ is the total points in the plot. These indices are computed within the time window defined as $\Omega_{t}:\;t--T\;\leq \;t_{i}\;\leq \;t$, where *T* was defined at 15 s, as per previous simulations (Candia‐Rivera et al., 2021, 2025). The computation step was defined by each heartbeat timing and the final time series were evenly interpolated at 4 Hz.

To statistically evaluate the couplings, we used Spearman correlation coefficient. Spearman *p*‐values were derived using a Student's *t* distribution approximation. Time lag between the time series was considered in the range of −20 to 20 s. Significance for correlations was set to α = 0.001. For discerning between the two experimental conditions, we used a two‐sided non‐parametric Wilcoxon rank sum test for unpaired samples. Since this is an exploratory study with respect to differences between pregnancy and labour, Wilcoxon's significance was defined at α = 0.01, as per the Bonferroni correction for the number of cardiac measures compared.

All physiological data used in this study are publicly available (Matonia et al., 2020). Codes implementing the methods of this study are available at https://github.com/diegocandiar/robust_hrv.

## 3 RESULTS

**FIGURE 2:**
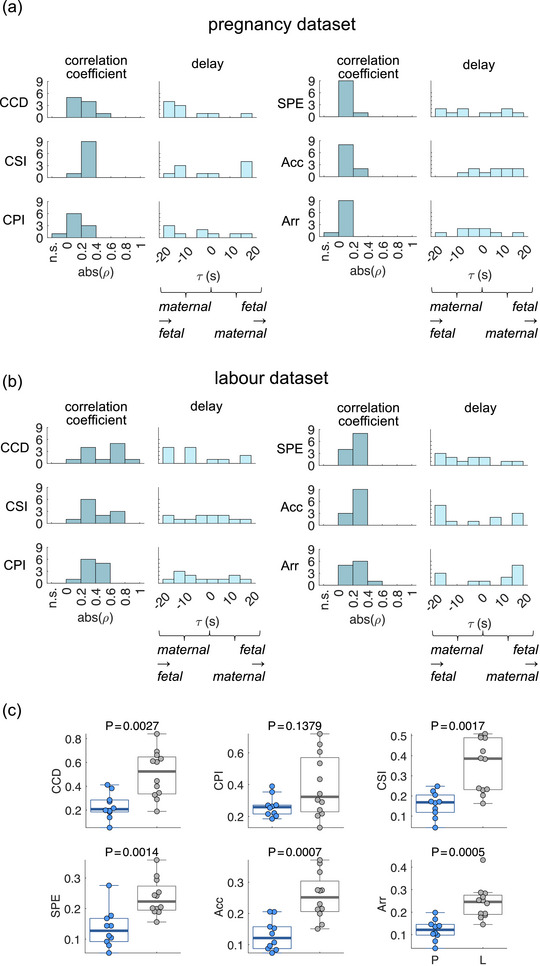
Maternal–fetal cardiac interaction results. (a,b) Histograms of the correlation coefficients (absolute values), from the significant Spearman correlations (*P *< 0.001). n.s., non‐significant correlation. Results are displayed for the six measures of heart rate and rhythm studied: baseline CCD, CSI, CPI, heart rate acceleration–deceleration balance (Acc), heart rate arrhythmic behaviour (Arr), and entropy (E). Next to each correlation coefficient histogram are displayed the respective time delay histograms, indicative of the delay in which the maximum absolute correlation coefficient was obtained. A negative delay indicates that maternal changes precede fetal changes, and vice versa for the positive ones. Results displayed in (a) correspond to the pregnancy dataset, and in (b) correspond to the labour dataset. (c) Results on the comparison between correlation coefficients obtained in the pregnancy (P) and labour (L) datasets. *P*‐values correspond to the unpaired Wilcoxon rank sum test. Significance for the Wilcoxon test is defined at α = 0.01, as per Bonferroni correction. CCD, cardiac cycle duration; CPI, cardiac parasympathetic index; CSI, cardiac sympathetic index.

We explored the multifaceted cardiac couplings through different measures of heart rate and rhythm from maternal and fetal cardiac activities. First, we verified the correlation between the measures of heart rate and rhythm of each maternal–fetal pair using Spearman's correlation. Figure 2a shows the results for the pregnancy dataset. Most descriptors were correlated in all maternal–fetal pairs (*P *< 0.001). No specific time lag interval showed predominance (time lags can be either positive or negative), indicating the potential for differing response times to a common stimulus or the existence of bilateral interactions, even though these were not directly controlled in this study. The predominance of low correlation coefficients may suggest the occurrence of brief episodes of coupling throughout the analysed recording (see Supporting information, Figures S1–S6). However, the potential presence of spurious correlations cannot be entirely ruled out. Figure 2b shows the results for the labour dataset, where all descriptors were correlated in all maternal–fetal pairs (*P *< 0.001). Similar to the pregnancy dataset, there was no predominance of a specific time lag interval, suggesting that the directionality of interactions is not necessarily modulated during labour, and it remains bilateral.

The correlation coefficient distributions showed higher values in the labour dataset compared to the pregnancy dataset. Figure 2c presents the statistical comparison between the pregnancy and labour datasets using unpaired Wilcoxon rank sum tests. We found that maternal–fetal cardiac couplings are stronger during labour compared to the resting state in pregnancy for most measures of heart rate and rhythm. Notably, the differences were greater in the measures derived from second‐order Poincaré plots, specifically in cardiac acceleration–deceleration balance, arrhythmic behaviour, and entropy. This suggests that maternal–fetal cardiac couplings are multifaceted, occurring beyond simple heart rate coupling.

**FIGURE 3:**
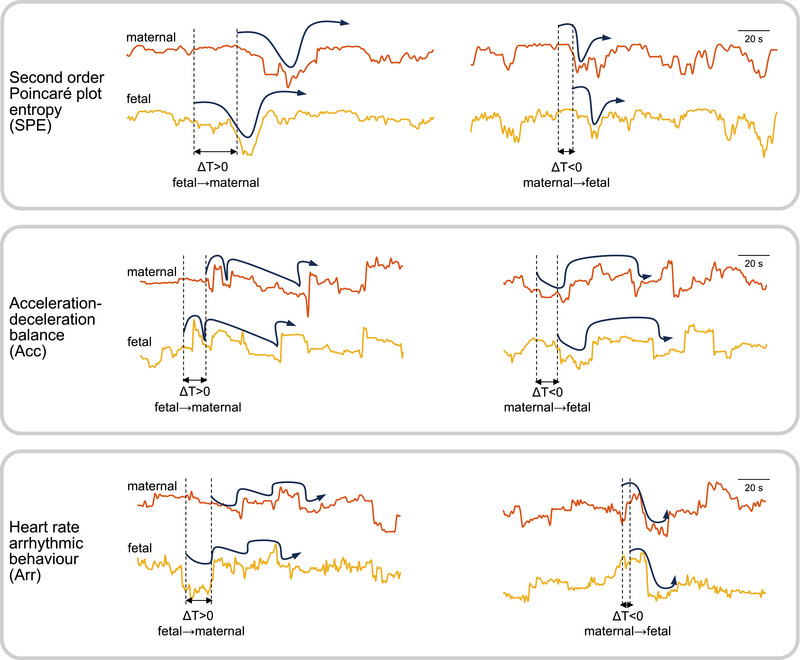
Examples of time delayed correlations between maternal and fetal cardiac activities. One example is displayed for each of three cardiac measures derived from the second‐order Poincaré plot, and for both maternal → fetal and fetal → maternal interactions.

Figure 3 illustrates examples of the diverse range of time delays and the associated directions in maternal–fetal cardiac interactions. The examples highlight the variability, from the measures of heart rate derived from second‐order Poincaré plots, in how the maternal heartbeat potentially influences the fetal heartbeat and vice versa. This diversity is indicative of the complex and dynamic nature of the physiological coupling between the mother and the fetus.

## 4 DISCUSSION

In this study, we investigated potential interactions between maternal and fetal cardiac autonomic activity using time‐varying measures of heart rate and rhythm derived from concurrent electrocardiogram recordings. Our main findings revealed episodes of coupling between maternal and fetal heart rate and rhythm measures. These couplings were characterized by delays in both directions, suggesting the possibility of bilateral interactions between maternal and fetal cardiac autonomic activities. Additionally, these interactions were greater during labour, suggesting the presence of co‐regulatory mechanisms. These results may suggest a potential active role of fetal physiology in the maternal inter‐organ communication, underscoring the importance of understanding the complexity of visceral oscillations in utero for healthy fetal development.

During pregnancy, the autonomic nervous system regulates physiological changes to support both the mother and the developing fetus (Ekholm & Erkkola, 1996). Sympathetic activity helps to manage the increased demand for oxygen and nutrients by raising heart rate and blood pressure, ensuring blood supply to the placenta and fetus. Conversely, parasympathetic activity promotes relaxation and conserves energy, aiding in digestion and stress reduction. Together, these activities maintain a fine balance, adapting to the dynamic needs of pregnancy and fostering a healthy environment for fetal development.

During labour and delivery, the nervous system plays a critical role through a combination of hormonal and neural mechanisms (Mastorakos & Ilias, 2003). Mechanisms operating within the hypothalamic–pituitary–adrenal axis that promote, for instance, the release of oxytocin, which stimulates uterine contractions, and ultimately affects the fetal heart as well. Sensory mechanisms in the uterus further contribute to promote the signalling that triggers the increase of oxytocin release in a positive feedback loop. Additionally, sympathetic activations modulate pain and stress responses until childbirth (Alehagen et al., 2005). These interactions ensure coordinated regulation during labour. In our results, the increased coupling of cardiac autonomic measures during labour suggests a potential engagement of both the sympathetic and parasympathetic nervous systems, underscoring the importance of maternal–fetal physiological crosstalk during this process. The high coupling of ongoing changes in cardiac entropy indicates dynamic fluctuations in cardiac activity, with both acceleration and deceleration phases. This could reflect the active and balanced involvement of both the sympathetic and parasympathetic nervous systems during these rapid changes.

Numerous methodologies and populations in studies of maternal–fetal cardiac coupling have been performed to date, showing that these interactions appear to occur bilaterally (Nichting et al., 2023). Existing literature has underscored changes in the maternal–fetal heart rate coupling as a function of physical activity (Van Leeuwen et al., 2014), stress (Lobmaier et al., 2020), respiration rate (Van Leeuwen et al., 2009) and pregnancy stage (Khandoker et al., 2016; Sletten et al., 2016; Wahbah et al., 2021). For instance, maternal to fetal interaction increases with gestational age, which has been hypothesized to relate to the fetal development in its auditory and autonomic nervous systems (Kisilevsky et al., 1992; Schneider et al., 2018), while fetal to maternal seems to be modulated by the maternal responsiveness to fetal movements (Entringer et al., 2010).

The mechanisms and the functions of these cardiac interactions remain unknown (Nichting et al., 2023). Among the potential pathways are nerve links through the placenta and neurotropic mechanisms (DiPietro et al., 2013; Khandoker et al., 2022; Owman et al., 1967). Pulse sensing can partially explain these dynamics (Van Leeuwen et al., 2009, 2014), for instance, by sensing the vibroacoustic effect triggered by changes in mechanical energy from the maternal vessels in a detectable frequency at fetus level. Pulse mechanosensation can also occur at the cellular level, through mechanosensitive ion channels (Jammal Salameh et al., 2024) and cilia (Djenoune et al., 2022), potentially enabling a faster relay of cardiac information and explaining the possibility of bilateral fetal–maternal interaction. Parallel modulation driven by maternal respiration can partially explain maternal–fetal cardiac couplings (DiPietro et al., 2021), where the visceral movements triggered by the maternal respiration could trigger changes in the fetal cardiac activity (DiPietro et al., 2023; Van Leeuwen et al., 2009). Indeed, this is a known effect in the context of cardiorespiratory couplings (Barbieri et al., 2002).

Maternal–fetal cardiac interaction assessments have a strong clinical potential, especially for the search of early biomarkers for specific developmental anomalies (Entringer et al., 2010; Kutlu et al., 2017; Moors et al., 2020). The understanding of visceral oscillations in utero can become paramount to asses early cognitive development (Corcoran et al., 2023), in particular when it is believed that physiological mechanisms are highly modulated by maternal psychological factors, such as the hypothalamic–pituitary–adrenal axis (Ochedalski & Lachowicz, 2004). From a more fundamental point of view, the understanding of an active role of early visceral oscillations could shed light on the early differentiation of the maternal–fetal interface (Junyent et al., 2024) and the mother's physiological adaptations (Hoekzema et al., 2017; Pritschet et al., 2024), in the context of inter‐organ communications.

Limitations of this study include sample size and lack of diversity of the population, in terms of gestational age or presence of pathologies. However, while other studies have highlighted the changes in maternal–fetal cardiac couplings during labour (Tepichín‐Castro et al., 2021), this is the first attempt at disentangling these couplings in different measures of heart rate and rhythm whose physiological meaning could be interpreted.

In light of the abundant evidence suggesting that observing simple heart rate couplings is not enough (Dipietro et al., 2004), other studies have implemented information theory‐based approaches to understand fetal–maternal cardiac couplings (Avci et al., 2018; Khandoker et al., 2019; Marzbanrad et al., 2015). Although information‐theoretic measures can offer valuable insights, by characterizing specific experimental conditions (Javorka et al., 2018), they often lack the direct physiological interpretability for a potential clinical translation (Huikuri et al., 2003). Our approach of disentangling different components of cardiac activity, describing the multifaceted nature of heart rate variability, is a compelling alternative to understand maternal–fetal couplings in different contexts.

It is important to note that the measured fetal–maternal cardiac interactions can stem from the physiological response to a specific stimulus, such as metabolic mechanisms (Clarke et al., 2024; Díaz et al., 2014), at different latencies between fetus and mother. During labour, contractions may affect the mother and fetus differently – through maternal discomfort and fetal hypoxia, respectively (Casati et al., 2014; Nichting et al., 2023; Sletten et al., 2016). The typical cardiac responses to contractions are reduced fetal heart rate and increased maternal heart rate. Therefore, the intensity of contractions may account for the seemingly stronger correlations between maternal and fetal heart rates during labour (CCD descriptor). However, this may not necessarily account for the other cardiac measures studied. Moreover, a key limitation remains with respect to the specificity of the proposed measures and whether they effectively capture sympathetic and parasympathetic dynamics.

Another important point is that different interactions can occur at varying time scales and in different directions within the time intervals studied. Because we did not conduct a time‐resolved analysis on the interactions, our results reflect the overall effect across the entire experimental condition, likely capturing the dominant interaction that repeats consistently at the same time scale across different segments. As the main objective of this study is to propose a time‐resolved measure of cardiac activities, the absence of a time‐resolved estimation of fetal–maternal cardiac interactions remains a limitation. This approach will be necessary in future research to better understand these dynamics in specific experimental conditions, including maternal physiological determinants, delivery complications or developmental pathologies. Finally, this study did not account for the specific states of the fetus. Given the use of short‐duration recordings, the heterogeneity of the datasets likely includes variations in fetal sleep and awake states.

### 4.1 Conclusion

We conclude that maternal–fetal cardiac interactions occur during late pregnancy and labour. These interactions are bilateral and multifaceted, indicating a potential ongoing presence of co‐regulatory mechanisms. However, the physiological underpinnings of maternal–fetal cardiac couplings need to be further investigated. More research is needed to clarify the physiological pathways and the specific role of the different types of couplings. This research agenda will ultimately improve the monitoring of fetal development, maternal–fetal well‐being assessments and abnormality detections.

## AUTHOR CONTRIBUTIONS

Diego Candia‐Rivera: Conceptualization, Methodology, Software, Validation, Formal analysis, Investigation, Writing—Original Draft, Writing—Review & Editing, Visualization, Funding acquisition. Mario Chavez: Methodology, Software, Supervision, Writing—Review & Editing. All authors have read and approved the final version of this manuscript and agree to be accountable for all aspects of the work in ensuring that questions related to the accuracy or integrity of any part of the work are appropriately investigated and resolved. All persons designated as authors qualify for authorship, and all those who qualify for authorship are listed.

## CONFLICT OF INTEREST

None declared.

## Supporting information



Supplementary Materials: Individual dyadic correlation plots

## Data Availability

The datasets used in this study come from an open access resource, as detailed in the original dataset publication (Matonia et al., 2020).
