# Supplementary material for: A method for dyadic cardiac rhythmicity analysis: Preliminary evidence on bilateral interactions in fetal–maternal cardiac dynamics
Source: Exp Physiol. 2025 Feb 21;110(8):1049–59. doi: 10.1113/EP092532 (PMC12314647; doi:10.1113/EP092532)
Supplement: Supplementary file 1 — Supplementary Materials: Individual dyadic correlation plots [file EPH-110-1049-s001.pdf]

Supplementary Material

A method for dyadic cardiac rhythmicity analysis: Preliminary evidence on bilateral interactions in fetal-maternal cardiac dynamics

Diego Candia-Rivera, Mario Chavez

Sorbonne Université, Paris Brain Institute (ICM), CNRS UMR7225, INSERM U1127, Hôpital de la Pitié Salpêtrière AP-HP, 75013, Paris, France.

* *Correspondence: diego.candia.r@ug.uchile.cl*

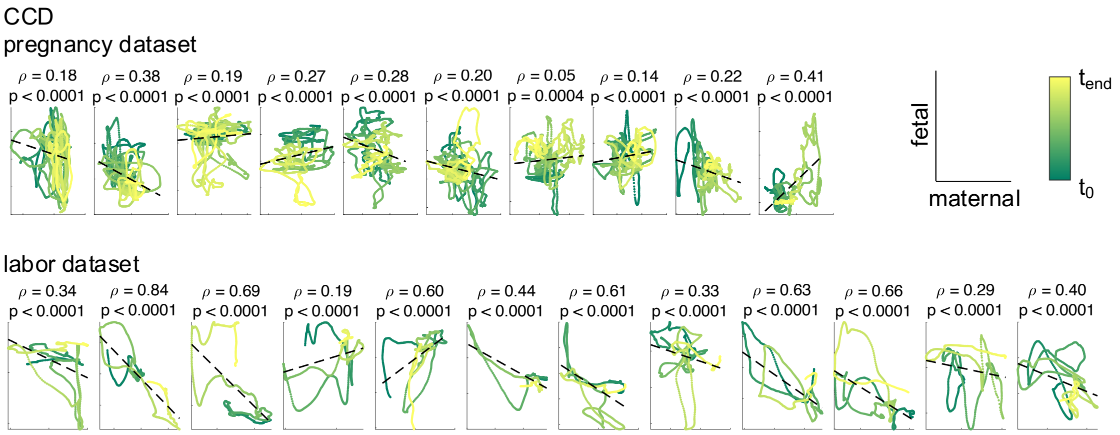

Figure S1. Scatter plots of Cardiac Cycle Duration (CCD) for each fetal-maternal pair, shown for both pregnancy and labor datasets. The correlation corresponds to the time delay that maximizes the absolute Spearman correlation coefficient within a time window of -20 to 20 seconds. Spearman correlation coefficients (ρ) and their respective p-values are displayed on each plot. Dashed lines represent the correlation trend, while the color bar indicates the time progression from the start to the end of the recording.

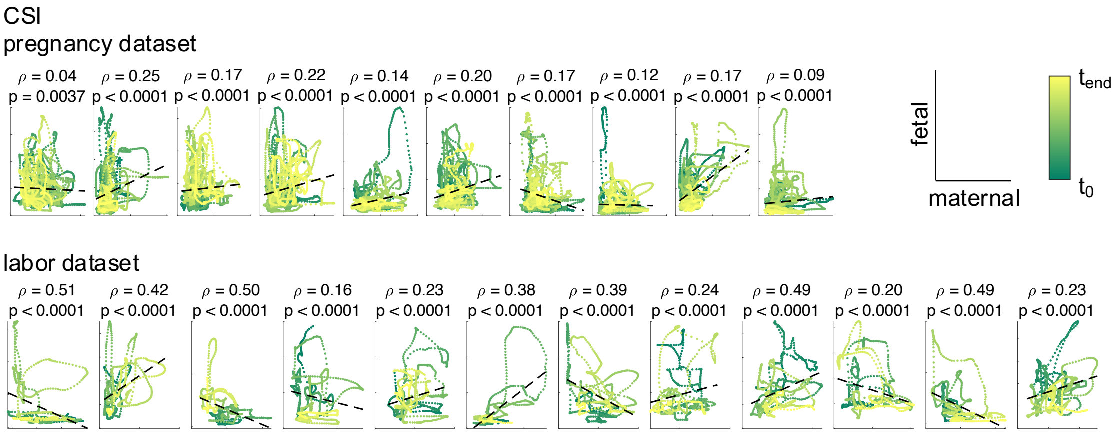

Figure S2. Scatter plots of Cardiac Sympathetic Index (CSI) for each fetal-maternal pair, shown for both pregnancy and labor datasets. The correlation corresponds to the time delay that maximizes the absolute Spearman correlation coefficient within a time window of -20 to 20 seconds. Spearman correlation coefficients (ρ) and their respective p-values are displayed on each plot. Dashed lines represent the correlation trend, while the color bar indicates the time progression from the start to the end of the recording.

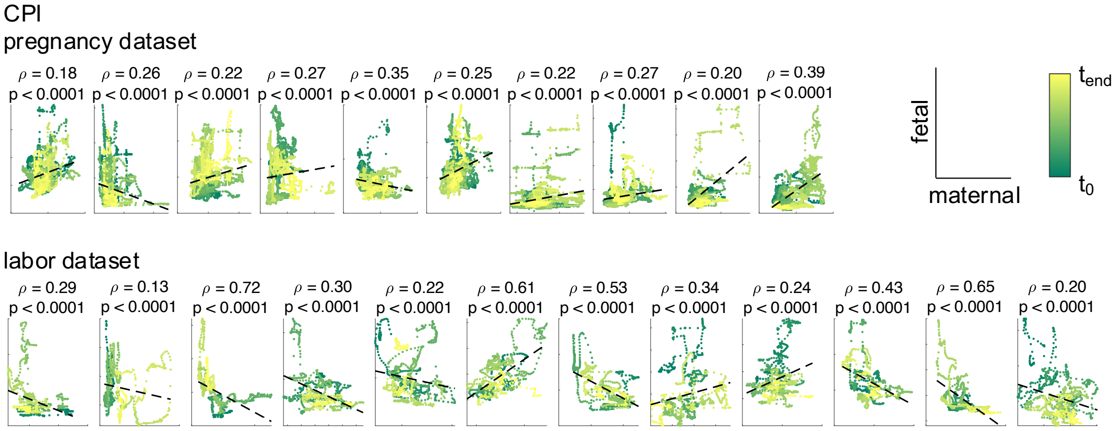

Figure S3. Scatter plots of Cardiac Parasympathetic Index (CPI) for each fetal-maternal pair, shown for both pregnancy and labor datasets. The correlation corresponds to the time delay that maximizes the absolute Spearman correlation coefficient within a time window of -20 to 20 seconds. Spearman correlation coefficients (ρ) and their respective p-values are displayed on each plot. Dashed lines represent the correlation trend, while the color bar indicates the time progression from the start to the end of the recording.

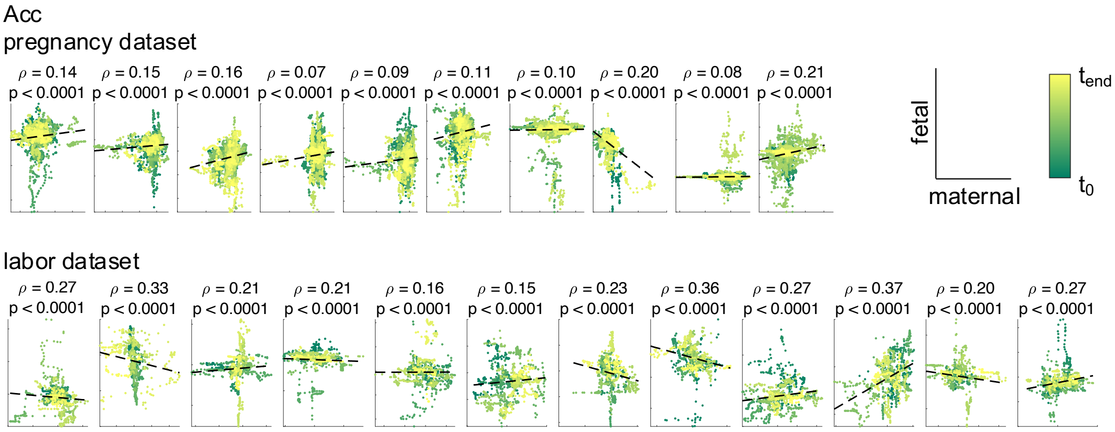

Figure S4. Scatter plots of Heart rate acceleration-deceleration balance (Acc) for each fetal-maternal pair, shown for both pregnancy and labor datasets. The correlation corresponds to the time delay that maximizes the absolute Spearman correlation coefficient within a time window of -20 to 20 seconds. Spearman correlation coefficients (ρ) and their respective p-values are displayed on each plot. Dashed lines represent the correlation trend, while the color bar indicates the time progression from the start to the end of the recording.

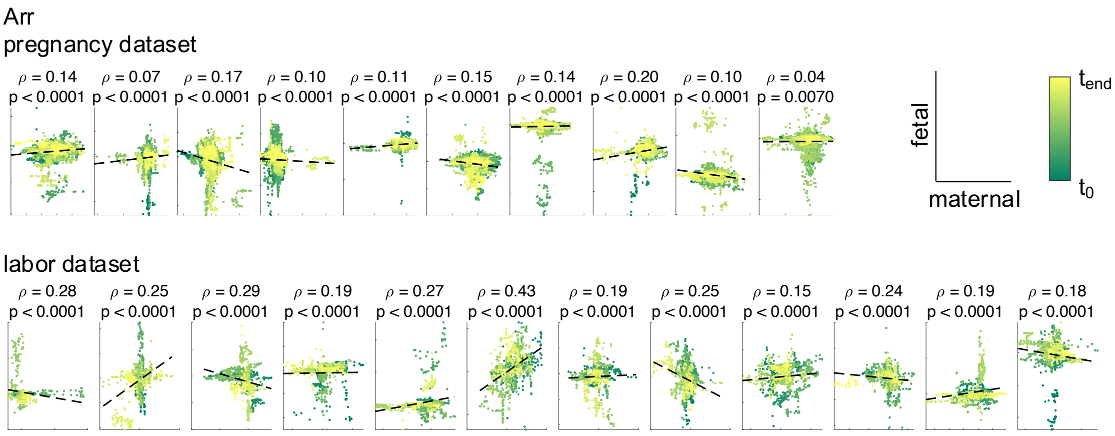

Figure S5. Scatter plots of Heart rate arrhythmic behavior (Arr) for each fetal-maternal pair, shown for both pregnancy and labor datasets. The correlation corresponds to the time delay that maximizes the absolute Spearman correlation coefficient within a time window of -20 to 20 seconds. Spearman correlation coefficients (ρ) and their respective p-values are displayed on each plot. Dashed lines represent the correlation trend, while the color bar indicates the time progression from the start to the end of the recording.

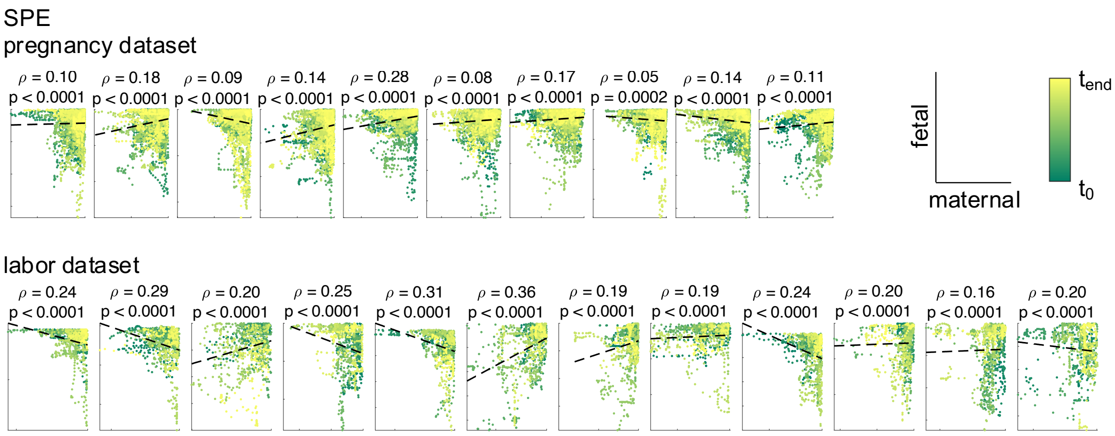

Figure S6. Scatter plots of second order Poincaré plot entropy (SPE) for each fetal-maternal pair, shown for both pregnancy and labor datasets. The correlation corresponds to the time delay that maximizes the absolute Spearman correlation coefficient within a time window of -20 to 20 seconds. Spearman correlation coefficients (ρ) and their respective p-values are displayed on each plot. Dashed lines represent the correlation trend, while the color bar indicates the time progression from the start to the end of the recording.
